# An Efficient Synthesis of Bis-indolylindane-1,3-diones, Indan-1,3-diones, and Indene-1,3(2H)-denies Using [Hbim]BF_**4**_ Ionic Medium

**DOI:** 10.1155/2013/528329

**Published:** 2013-12-16

**Authors:** Mohammad Reza Poor Heravi

**Affiliations:** Department of Chemistry, Payame Noor University, P.O. Box 19395-3697, Tehran, Iran

## Abstract

We prepared a brand new molecule in one step for the synthesis of bis-indolylindane-1,3-dione and indan-1,3-diones from the reaction of ninhydrin and 3 substituted/unsubstituted indoles using [Hbim]BF_4_ ionic liquid in excellent yields. The method was also used for the synthesis of novel indene-1,3(2H)-denies derivatives.

## 1. Introduction

In recent times, ionic liquids have gained recognition as possible environmentally benign alternatives to the more volatile organic solvents [1]. Ionic liquids possess many attractive properties, such as wide liquid range, negligible vapor pressure, ease of recyclability, high thermal stability, and good solvating ability in a wide range of substrates and catalysts, which alleviate some of the environmental issues. Their nonvolatile nature can reduce the emission of organic compounds and facilitate the separation of products and/or catalysts from the reaction solvents. Furthermore, ionic liquids are found to be an efficient reaction medium for the immobilization of transition metal-based catalysts, Lewis acids, and enzymes [2]. The hallmark of such ionic liquids is the ability to alter their properties as desired by manipulating their structure with respect to the choice of organic cation or anion and side chain attached to the organic cation. Important pharmaceuticals often possess heterocyclic moieties as their building blocks [3]. The extensive use of heterocyclic compounds in the pharmaceutical industry is perhaps attributable to the availability of ample range of reactions that facilitate subtle structural modifications in heterocyclic compounds [4–7]. Since indole and its derivatives possess various biological activities [8], development of new methodologies for the synthesis of indole derivatives, which will yield subsets of heterocycles having potentiality to serve as templates for new biologically active molecules, is of great importance.

In this context, we wish to describe a convenient and simple methodology for the synthesis of bis-indolylindane-1,3-dione (by reacting ninhydrin with 3 substituted/unsubstituted indoles), 2-(1′,3′-dihydro-1H-[2,3′]biindolyl-2′-ylidene)-indan-1,3-diones, indene-1,3(2H)-denies (from the reaction of ninhydrin, 1,2-phenylendiamine, and indole), and 2,2-bis(4-(dimethylamino)phenyl)-1H-indene-1,3(2H)-diones (from the reaction of ninhydrin with N,N-dimethylaniline). The reactions were carried out using [Hbim]BF_4_ ionic liquid as green solvent. The novelty of the methodology lies in its eco-friendly operation, the formation of structurally unique molecules, short reaction time, and excellent yield.

## 2. Experimental

### 2.1. General

All reagents were purchased from Merck and Aldrich and used without further purification. The ionic liquid, [Hbim][BF]_4_, was synthesized by the method reported in [9]. Melting points were determined using a Linkman HF591 heating stage, used in conjunction with a TC92 controller, and reuncorrected. NMR spectra were recorded using a Bruker DRX500 machine at room temperature. ^1^H and ^13^C NMR spectra were measured using deuterochloroform as solvent, and chemical shifts were measured relatively to residual solvent or CFCl_3_ as an internal standard for ^19^F NMR and are expressed in parts per million (*δ*). Mass spectra were obtained using a Micro Mass LCT machine in ES or EI mode. Infrared spectra were measured on a Perkin Elmer Paragon 100 FT-IR spectrometer. Analytical thin layer chromatography (TLC) for monitoring reactions was performed using Merck 0.2 mm silica gel 60 f-254 Al-plates.

### 2.2. General Procedure for the Synthesis of Bis-indolylindane-1,3-dione, 2-(1′,3′-Dihydro-1H-[2,3′]biindolyl-2′-ylidene)-indan-1,3-diones, Indene-1,3(2H)-denies, and 2,2-Bis(4-(dimethylamino)phenyl)-1H-indene-1,3(2H)-diones

1 mmol ninhydrin (**1**) and 2 mmol indole derivatives **2(a–e)** (for the synthesis **3(a–e)**), 1 mmol ninhydrin (**1**) 1 mmol 1,2-phenylenediamine derivatives **4(a–c)**, and 2 mmol indole derivatives **2(a–d)** (for the synthesis **6aa–6ae, 6ba–6be, 6ca–6ce**) or 1 mmol ninhydrin (**1**), 2 mmol N,N-dimethylaniline **7(a–c)** (for the synthesis **8(a–c)**) were added to a 20 mL round bottom flask containing 2 mL [Hbim]BF_4_. The mixture was stirred at room temperature 25°C for appropriate time (monitored by TLC). After completion of the reaction, the reaction mixture was added with 5 mL water (IL is soluble in water). The precipitate was collected by filtration and purified by crystallization from chloroform/methanol to afford pure products. The filtrate was concentrated under reduced pressure and dried at 100°C to recover the ionic liquid for subsequent use. 

Spectroscopic data of new products are given below.

#### 2.2.1. 2,2-Bis(5-fluoro-1H-indol-3-yl)-1H-indene-1,3(2H)-dione **3b** (Table 1, Entry 2)

Yellow prisms, mp = 121–123°C, IR (KBr): *ν*_max⁡_ = 3399, 1706, 1254, 755 cm^−1^; ^1^H NMR (500 MHz, DMSO-*d*_6_): *δ* = 7.28 (s, 2H), 7.38 (m, 2H), 7.41 (m, 2H), 7.45 (s, 2H), 7.76 (m, 1H), 7.87 (m, 1H), 8.11 (m, 1H), 8.25 (m, 1H), 12.54 (s, 2H, −NH) ppm; ^13^C NMR (125 MHz, DMSO-*d*_6_): *δ* = 58.1 (C), 111.7 (2 × C), 112.5 (2 × C), 115.1 (2 × CH), 125.9 (2 × CH), 127.2 (4 × CH), 128.8 (2 × CH), 129.5 (2 × C), 132.5 (2 × CH), 137.8 (2 × C), 151.6 (d, ^1^ *J*_CF_ = 250.3 Hz, 2 × C–F), 197.8 (2 × CO) ppm; ^19^F NMR (DMSO-*d*_6_, 470 MHz): −73.25; MS (EI), *m*/*z* (%) = 412 (M^+^, 27), 144 (65); HRMS (EI) Found: M^+^, 412.1008. C_25_H_15_F_3_N_2_S requires M^+^, 412.1011; Anal Calcd. for C_25_H_15_F_3_N_2_S, C, 72.81; H, 3.42; N, 6.79. Found: C, 72.90; H, 3.41; N, 6.71.

#### 2.2.2. 11,11-Bis-(5-fluoro-1H-indol-3-yl)-11H-indeno[1,2-b]quinoxaline **5ab** (Table 2, Entry 2)

Yellow prisms, mp = 224–226°C, IR (KBr): *ν*_max⁡_ = 3408, 1459, 1121, 763 cm^−1^; ^1^H NMR (500 MHz, DMSO-*d*_6_): *δ* = 7.11 (s, 2H), 7.31 (m, 2H), 7.52 (m, 6H), 7.85 (d, 1H, *J* = 7.6 Hz), 8.03 (d, 1H, *J* = 8.5 Hz), 8.11 (s, 2H), 8.19 (d, 1H, *J* = 8.5 Hz), 8.62 (d, 1H, *J* = 7.4 Hz), 12.45 (s, 2H, −NH) ppm; ^13^C NMR (125 MHz, DMSO-*d*_6_): *δ* = 55.2 (C), 112.4 (2 × C), 116.8 (2 × CH), 117.9 (2 × C), 123.7 (CH), 125.8 (CH), 126.7 (CH), 127.5 (CH), 128.0 (CH), 129.2 (2 × C), 129.6 (CH), 130.2 (CH), 130.8 (CH), 130.9 (CH), 132.2 (CH), 133.2 (CH), 133.9 (CH), 137.5 (C), 138.2 (C), 138.9 (C), 141.2 (C), 151.8 (d, ^1^ *J*_CF_ = 250.1 Hz, 2 × C–F), 154.2 (C), 168.5 (C) ppm; ^19^F NMR (DMSO-*d*_6_, 470 MHz): −78.45; MS (EI), *m*/*z* (%) = 484 (M^+^, 18), 184 (55); HRMS (EI) Found: M^+^, 485.1507. C_31_H_18_F_2_N_4_ requires M^+^, 484.1501; Anal Calcd. for C_31_H_18_F_2_N_4_, C, 76.85; H, 3.74; N, 11.56. Found: C, 76.94; H, 3.61; N, 11.60.

#### 2.2.3. 11,11-Bis(2-methyl-1H-indol-3-yl)-11H-indeno[1,2-b]quinoxaline **5ad** (Table 2, Entry 4)

Green prisms, mp = 195–197°C, IR (KBr): *ν*_max⁡_ = 3416, 2954, 1468, 763 cm^−1^; ^1^H NMR (500 MHz, DMSO-*d*_6_): *δ* = 2.58 (s, 6H, CH_3_), 6.98 (s, 2H), 7.02 (t, 2H, *J* = 7.5 Hz), 7.12 (t, 2H, *J* = 7.3 Hz), 7.31 (m, 2H), 7.54 (t, 1H, *J* = 7.5 Hz), 7.68 (m, 4H), 7.94 (t, 1H, *J* = 7.5 Hz), 8.01 (m, 2H), 8.09 (m, 2H), 8.12 (d, 1H, *J* = 8.2 Hz), 8.41 (d, 1H, *J* = 8.5 Hz), 8.41 (d, 1H, *J* = 7.5 Hz) ppm; ^13^C NMR (125 MHz, DMSO-*d*_6_): *δ* = 32.6 (CH_3_), 33.1 (CH_3_), 54.2 (C), 110.1 (2 × CH), 115.4 (2 × C), 117.9 (2 × CH), 122.5 (2 × CH), 123.4 (2 × CH), 124.2 (2 × CH), 126.2 (C), 126.7 (CH), 128.0 (CH), 128.6 (CH), 128.9 (CH), 129.4 (C), 129.5 (2 × CH), 130.4 (C), 137.2 (C), 137.8 (2 × C), 140.0 (C), 141.2 (C), 152.1 (2 × C), 152.4 (C), 159.4 (C) ppm; MS (EI), *m*/*z* (%) = 476 (M^+^, 9), 210 (43); HRMS (EI) Found: M^+^, 476.2008. C_33_H_24_N_4_ requires M^+^, 476.2001; Anal Calcd. for C_33_H_24_N_4_, C, 83.17; H, 5.08; N, 11.76. Found: C, 83.14; H, 5.11; N, 11.81.

#### 2.2.4. 11,11-Bis(5-fluoro-1H-indol-3-yl)-7,8-dimethyl-11H-indeno[1,2-b]quinoxaline **5bb** (Table 2, Entry 7)

Brown needles, mp = 210–212°C, IR (KBr): *ν*_max⁡_ = 3430, 2935, 1454, 746 cm^−1^; ^1^H NMR (500 MHz, DMSO-*d*_6_): *δ* = 3.25 (s, 6H, CH_3_), 6.81 (s, 2H), 6.91 (m, 4H), 7.21 (m, 2H), 7.55 (d, 1H, *J* = 7.5 Hz), 7.65 (d, 1H, *J* = 8.5 Hz), 7.85 (s, 2H), 7.92 (d, 1H, *J* = 8.5 Hz), 8.12 (d, 1H, *J* = 7.5 Hz), 11.86 (s, 2H, −NH) ppm; ^13^C NMR (125 MHz, DMSO-*d*_6_): *δ* = 35.9 (2 × CH_3_), 54.7 (C), 110.4 (2 × CH), 114.8 (2 × C), 116.9 (2 × CH), 122.5 (2 × CH), 124.8 (CH), 125.8 (CH), 126.5 (CH), 127.5 (2 × C), 128.2 (CH), 129.0 (CH), 130.1 (CH), 130.4 (CH), 131.9 (CH), 131.8 (C), 132.2 (C), 133.4 (C), 135.5 (C), 137.2 (2 × C), 139.4 (C), 140.2 (C), 152.8 (d, ^1^ *J*_CF_ = 251.6 Hz, 2 × C–F), 155.4 (C), 169.7 (C) ppm; ^19^F NMR (DMSO-*d*_6_, 470 MHz): −76.78; MS (EI), *m*/*z* (%) = 512 (M^+^, 15), 252 (51); HRMS (EI) Found: M^+^, 512.1804. C_33_H_22_F_2_N_4_ requires M^+^, 512.1807; Anal Calcd. for C_33_H_22_F_2_N_4_, C, 77.33; H, 4.33; N, 10.93. Found: C, 77.41; H, 4.31; N, 11.01.

#### 2.2.5. 11,11-Bis(5-bromo-1H-indol-3-yl)-7,8-dimethyl-11H-indeno[1,2-b]quinoxaline **5bc** (Table 2, Entry 8)

Green needles, mp = 219–221°C, IR (KBr): *ν*_max⁡_ = 3427, 2987, 1472, 761 cm^−1^; ^1^H NMR (500 MHz, DMSO-*d*_6_): *δ* = 2.86 (s, 6H, CH_3_), 7.14 (s, 2H), 7.31 (m, 2H), 7.51 (m, 6H), 7.84 (d, 1H, *J* = 7.2 Hz), 7.86 (d, 1H, *J* = 8.3 Hz), 7.98 (s, 2H), 8.14 (d, 1H, *J* = 8.3 Hz), 8.24 (d, 1H, *J* = 7.2 Hz), 12.24 (s, 2H, −NH) ppm; ^13^C NMR  (125 MHz, DMSO-*d*_6_): *δ* = 37.2 (2 × CH_3_), 55.7 (C), 111.4 (2 × C), 115.7 (2 × CH), 117.5 (2 × C), 122.6 (2 × CH), 125.6 (CH), 126.6 (CH), 126.5 (CH), 127.5 (2 × C), 128.2 (CH), 129.0 (CH), 130.1 (CH), 130.4 (CH), 131.9 (CH), 128.5 (CH), 131.2 (2 × C), 131.6 (CH), 137.5 (2 × C), 138.4 (C), 141.2 (C), 143.8 (2 × C), 153.6 (C), 167.2 (C) ppm; MS (EI), *m*/*z* (%) = 634 (M^+^, 25), 384 (75); HRMS (EI) Found: M^+^, 632.0215. C_33_H_22_BrN_4_ requires M^+^, 632.0211; Anal Calcd. for C_33_H_22_BrN_4_, C, 62.48; H, 3.50; N, 8.83. Found: C, 62.51; H, 3.41; N, 8.89.

#### 2.2.6. 7,8-Dichloro-11,11-di(1H-indol-3-yl)-11H-indeno[1,2-b]quinoxaline **5ca** (Table 2, Entry 11)

Yellow needles, mp = 228–230°C, IR (KBr): *ν*_max⁡_ = 3427, 1438, 747 cm^−1^; ^1^H NMR (500 MHz, DMSO-*d*_6_): *δ* = 6.72 (s, 2H), 6.93 (m, 2H), 7.06 (m, 2H), 7.32 (m, 4H), 7.64 (m, 2H), 7.75 (d, 1H, *J* = 7.4 Hz), 7.96 (d, 1H, *J* = 7.4 Hz), 8.09 (s, 1H), 8.14 (d, 1H, *J* = 8.3 Hz), 12.24 (s, 2H, −NH) ppm; ^13^C NMR (125 MHz, DMSO-*d*_6_): *δ* = 53.7 (C), 109.4 (2 × CH), 115.4 (2 × C), 119.4 (2 × CH), 122.7 (CH), 125.9 (CH), 126.8 (2 × CH), 127.5 (2 × C), 127.9 (2 × CH), 1285 (CH), 129.4 (CH), 130.2 (CH), 130.8 (CH), 131.5 (2 × CH), 131.8 (C), 130.9 (C), 137.5 (2 × C), 138.8 (C), 140.7 (C), 142.8 (2 × C), 158.6 (C), 166.2 (C) ppm; MS (EI), *m*/*z* (%) = 517 (M^+^, 22), 257 (65); HRMS (EI) Found: M^+^, 516.0903. C_31_H_18_Cl_2_N_4_ requires M^+^, 516.0908; Anal Calcd. for C_31_H_18_Cl_2_N_4_, C, 71.96; H, 13.70; N, 10.83. Found: C, 71.89; H, 13.61; N, 10.89.

#### 2.2.7. 7,8-Dichloro-11,11-bis(5-fluoro-1H-indol-3-yl)-11H-indeno[1,2-b]quinoxaline **5cb** (Table 2, Entry 12)

Green needles, mp = 199–201°C, IR (KBr): *ν*_max⁡_ = 3435, 1452, 729 cm^−1^; ^1^H NMR (500 MHz, DMSO-*d*_6_): *δ* = 6.80 (s, 2H), 7.01 (m, 2H), 7.12 (m, 2H), 7.42 (m, 3H), 7.56 (m, 2H), 7.63 (d, 1H, *J* = 7.5 Hz), 7.89 (d, 1H, *J* = 7.5 Hz), 8.10 (s, 1H), 12.37 (s, 2H, −NH) ppm; ^13^C NMR (125 MHz, DMSO-*d*_6_): *δ* = 53.5 (C), 107.9 (2 × CH), 113.6 (2 × C), 117.7 (CH), 121.7 (CH), 123.9 (CH), 124.7 (2 × CH), 126.7 (2 × C), 128.3 (2 × CH), 128.8 (CH), 129.0 (CH), 131.2 (CH), 131.8 (CH), 132.5 (2 × CH), 133.5 (C), 134.8 (C), 136.9 (2×C), 137.8 (2 × C), 141.7 (C), 144.6 (2 × C), 156.9 (d, ^1^ *J*_CF_ = 253.1 Hz, 2 × C–F), 168.0 (C) ppm; ^19^F NMR (DMSO-*d*_6_, 470 MHz): −73.68; MS (EI), *m*/*z* (%) = 552 (M^+^, 12), 292 (65); HRMS (EI) Found: M^+^, 552.071012. C_31_H_16_Cl_2_F_2_N_4_ requires M^+^, 552.0710; Anal Calcd. for C_31_H_16_Cl_2_F_2_N_4_, C, 67.28; H, 2.91; N, 10.12. Found: C, 67.34; H, 2.98; N, 10.21.

#### 2.2.8. 11,11-Bis(5-bromo-1H-indol-3-yl)-7,8-dichloro-11H-indeno[1,2-b]quinoxaline **5cc** (Table 2, Entry 13)

Green needles, mp = 223–225°C, IR (KBr): *ν*_max⁡_ = 3425, 1434, 739 cm^−1^; ^1^H NMR (500 MHz, DMSO-*d*_6_): *δ* = 6.69 (s, 2H), 6.89 (m, 2H), 6.89 (m, 2H), 7.12 (m, 3H), 7.35 (m, 2H), 7.56 (d, 1H, *J* = 7.2 Hz), 7.69 (d, 1H, *J* = 7.2 Hz), 8.05 (s, 1H), 12.22 (s, 2H, −NH) ppm; ^13^C NMR (125 MHz, DMSO-*d*_6_): *δ* = 54.7 (C), 111.2 (2 × CH), 114.6 (2 × C), 118.8 (CH), 120.8 (CH), 122.7 (CH), 123.4 (2 × CH), 125.6 (2 × C), 126.8 (2 × CH), 127.6 (CH), 128.2 (CH), 130.2 (CH), 131.0 (CH), 133.5 (CH), 133.8 (C), 135.4 (C), 136.4 (2 × C), 137.4 (2 × C), 140.6 (C), 143.8 (2 × C), 155.6 (d, 2 × C), 169.4 (C) ppm; MS (EI), *m*/*z* (%) = 675 (M^+^, 11), 415 (34); HRMS (EI) Found: M^+^, 671.9112. C_31_H_16_Br_2_Cl_2_N_4_ requires M^+^, 671.9110; Anal Calcd. for C_31_H_16_Br_2_Cl_2_N_4_, C, 55.14; H, 2.39; N, 8.30. Found: C, 55.23; H, 2.38; N, 8.33.

#### 2.2.9. 7,8-Dichloro-11,11-bis(2-methyl-1H-indol-3-yl)-11H-indeno[1,2-b]quinoxaline **5cd** (Table 2, Entry 14)

Yellow needles, mp = 253–255°C, IR (KBr): *ν*_max⁡_ = 3434, 2982, 1456, 740 cm^−1^; ^1^H NMR (500 MHz, DMSO-*d*_6_): *δ* = 2.32 (s, 3H, CH_3_), 2.42 (s, 3H, CH_3_), 6.89 (s, 2H), 6.91 (m, 2H), 7.01 (m, 2H), 7.21 (m, 3H), 7.30 (m, 2H), 7.46 (d, 1H, *J* = 7.3 Hz), 7.51 (d, 1H, *J* = 7.3 Hz), 8.12 (s, 1H), 12.43 (s, 2H, −NH) ppm; ^13^C NMR (125 MHz, DMSO-*d*_6_): *δ* = 21.0 (CH_3_), 21.6 (CH_3_), 55.1 (C), 111.2 (2 × CH), 114.6 (2 × C), 118.8 (CH), 120.8 (CH), 122.7 (CH), 123.4 (2 × CH), 125.6 (2 × C), 126.8 (2 × CH), 127.6 (CH), 128.2 (CH), 130.2 (CH), 131.0 (CH), 133.5 (CH), 133.8 (C), 135.4 (C), 136.4 (2 × C), 137.4 (2 × C), 140.6 (C), 143.8 (2 × C), 155.6 (d, 2 × C), 169.4 (C) ppm; MS (EI), *m*/*z* (%) = 545 (M^+^, 10), 285 (35); HRMS (EI) Found: M^+^, 544.1200. C_33_H_22_Cl_2_N_4_ requires M^+^, 544.1205; Anal Calcd. for C_33_H_22_Cl_2_N_4_, C, 72.66; H, 4.07; N, 10.27. Found: C, 72.69; H, 4.08; N, 10.33.

#### 2.2.10. 2,2-Bis(4-(dimethylamino)phenyl)-1H-indene-1,3(2H)-dione **7a** (Table 3, Entry 1)

Brown needles, mp = 211–213°C, IR (KBr): *ν*_max⁡_ = 3056, 2984, 1715, 1625, 1451, 749 cm^−1^; ^1^H NMR (500 MHz, DMSO-*d*_6_): *δ* = 3.20 (s, 6H, 2 × CH_3_), 3.35 (s, 6H, 2 × CH_3_), 6.72 (d, 4H), 6.98 (m, 4H), 8.21 (d, 2H, *J* = 7.5 Hz), 8.13 (d, 2H, *J* = 7.5 Hz) ppm; ^13^C NMR (125 MHz, DMSO-*d*_6_): *δ* = 22.3 (2 × CH_3_), 23.1 (2 × CH_3_), 80.1 (C), 110.2 (4 × CH), 113.4 (2 × C), 114.8 (2 × CH), 115.7 (4 × CH), 117.6 (2 × CH), 145.2 (2C), 196.8 (2C) ppm; MS (EI), *m*/*z* (%) = 384 (M^+^, 15), 144 (26); HRMS (EI) Found: M^+^, 384.1819. C_25_H_24_N_2_O_2_ requires M^+^, 384.1821; Anal Calcd. for C_25_H_24_N_2_O_2_, C, 78.10; H, 6.29; N, 7.29. Found: C, 72.69; H, 4.08; N, 10.33.

#### 2.2.11. 2,2-Bis(4-(dimethylamino)-3-methylphenyl)-1H-indene-1,3(2H)-dione **7b** (Table 3, Entry 2)

Yellow needles, mp = 231–233°C, IR (KBr): *ν*_max⁡_ = 3050, 1705, 1456, 745 cm^−1^; ^1^H NMR (500 MHz, DMSO-*d*_6_): *δ* = 2.65 (s, 3H, CH_3_), 3.20 (s, 6H, 2 × CH_3_), 3.35 (s, 6H, 2 × CH_3_), 6.72 (d, 3H), 6.98 (m, 3H), 8.21 (d, 2H, *J* = 7.5 Hz), 8.13 (d, 2H, *J* = 7.5 Hz) ppm; ^13^C NMR (125 MHz, DMSO-*d*_6_): *δ* = 20.4 (CH_3_), 22.3 (2 × CH_3_), 23.1 (2 × CH_3_), 80.1 (C), 110.2 (3 × CH), 113.4 (3 × C), 114.8 (3 × CH), 115.7 (3 × CH), 117.6 (2 × CH), 145.2 (2C), 196.8 (2C) ppm; MS (EI), *m*/*z* (%) = 412 (M^+^, 17), 172 (35); HRMS (EI) Found: M^+^, 412.2209. C_27_H_28_N_2_O_2_ requires M^+^, 412.2211; Anal Calcd. for C_27_H_28_N_2_O_2_, C, 78.61; H, 6.84; N, 6.79. Found: C, 72.70; H, 6.90; N, 6.81.

#### 2.2.12. 2,2-Bis(3-chloro-4-(dimethylamino)phenyl)-1H-indene-1,3(2H)-dione **7c** (Table 3, Entry 3)

Yellow needles, mp = 225–227°C, IR (KBr): *ν*_max⁡_ = 3085, 1712, 1456, 729 cm^−1^; ^1^H NMR (500 MHz, DMSO-*d*_6_): *δ* = 3.23 (s, 6H, 2 × CH_3_), 3.29 (s, 6H, 2 × CH_3_), 6.81 (d, 3H), 6.92 (m, 3H), 8.04 (d, 2H, *J* = 7.2 Hz), 8.15 (d, 2H, *J* = 7.2 Hz) ppm; ^13^C NMR (125 MHz, DMSO-*d*_6_): *δ* = 22.3 (2 × CH_3_), 23.1 (2 × CH_3_), 80.1 (C), 110.2 (3 × CH), 113.4 (3 × C), 114.8 (3 × CH), 115.7 (3 × CH), 117.6 (2 × CH), 145.2 (2C), 196.8 (2C) ppm; MS (EI), *m*/*z* (%) = 453 (M^+^, 14), 213 (75); HRMS (EI) Found: M^+^, 412.2209. C_25_H_22_Cl_2_N_2_O_2_ requires M^+^, 412.2211; Anal Calcd. For C_25_H_22_Cl_2_N_2_O_2_, 66.23; H, 4.89; N, 6.18. Found: C, 66.27; H, 4.91; N, 6.14.

## 3. Results and Discussions

**Scheme 1:**
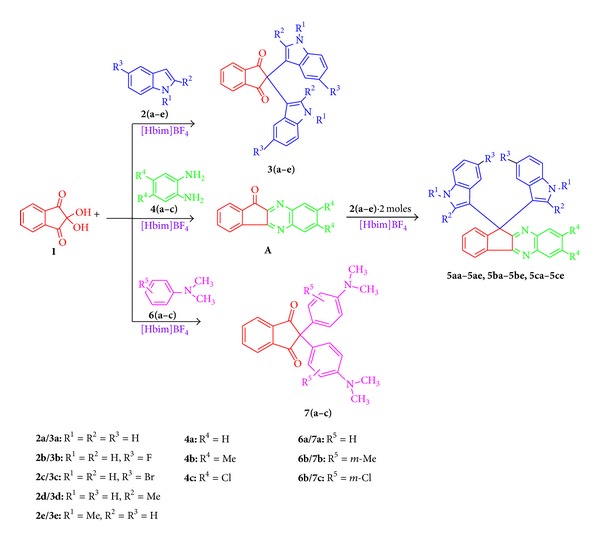
Synthesis of bis-indolylindane-1,3-dione, 2-(1′,3′-dihydro-1H-[2,3′]biindolyl-2′-ylidene)-indan-1,3-diones, **5**, and **7** in the presence of [Hbim]BF_4_ ionic medium.

With an ever increasing quest for the exploration of newer reactions in ionic liquids, the ionic liquid plays the dual role of solvent and promoter. Herein, we wish to report, for the first time, the use of [Hbim]BF_4_ ionic liquid as novel and recyclable polar reaction media for the synthesis of bis-indolylindane-1,3-dione, 2-(1′,3′-dihydro-1H-[2,3′]biindolyl-2′-ylidene)-indan-1,3-diones, and 2,2-bis(4-(dimethylamino)phenyl)-1H-indene-1,3(2H)-denies (Scheme 1).

First, 1 mmol ninhydrin (**1**) and 2 mmol different substituted indole derivatives (**2a–e**) were added to a 20 mL round bottom flask containing 2 mL [Hbim]BF_4_ ionic media. The resulting mixture stirred the appropriate time to afford his-indolylindane-1,3-dione, 2-(1′,3′-dihydro-1H-[2,3′]biindolyl-2′-ylidene)-indan-1,3-diones **3(a–e)** in excellent yield (Table 1). Differently substituted indole derivatives (**2a–e**) were reacted with ninhydrin (**1**). Of these, 5-fluoro (**2b**), 5-bromo (**2c**), 2-methyl (**2d**), 1-methyl (**2e**) indoles reacted smoothly to produce novel bis-indolylindane-1,3-dione, 2-(1′,3′-dihydro-1H-[2,3′]biindolyl-2′-ylidene)-indan-1,3-diones (Table 1, entries 2–5). The characteristic quaternary carbon signals **3(a–e)** clearly indicate the attachment of two indole moieties at C-2 of ninhydrin.

Next, I attempted to synthesize novel indene-1,3(2H)-denies reaction of ninhydrin (**1**) with 1,2-phenylenediamine **4(a–c)** and indole **2(a–e)** derivatives under the same reaction condition (Scheme 1). Interestingly, a variety of indoles including N-1, C-2, and C-6 substituted indoles participated well in this reaction and gave the corresponding products in excellent yield. As seen, indoles carrying electron-donating substituent act well in this reaction conditions (Table 2, entries 6–15).

Reaction ninhydrin (**1**, 1 mmol) and different substituted N,N-dimethyl aniline **6(a–c) **went smoothly in the ionic liquid [Hbim]BF_4_ under solvent free conditions to afford the corresponding products **7(a–c)** in high yields (Table 3, entries 1–3).

**Scheme 2:**
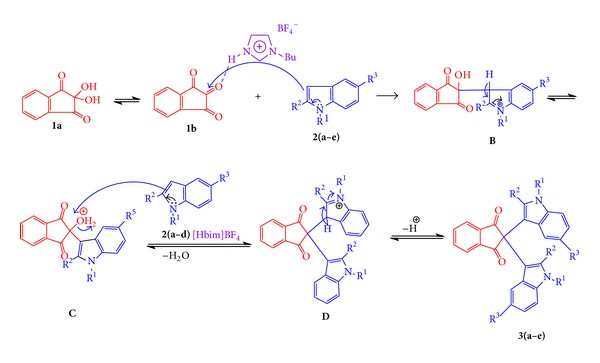
Plausible mechanism synthesis of bis-indolylindane-1,3-dione, 2-(1′,3′-dihydro-1H-[2,3′]biindolyl-2′-ylidene)-indan-1,3-diones, in the presence of [Hbim]BF_4_ ionic medium.

Ninhydrin is in equilibrium with indane-1,2,3-trione (**1b**). The nucleophilic substitution at C-3 of indole, produced intermediate **B**, via 1,3-migration hydrogen and aromatization of the indole ring produced **C** intermediate, which was attacked by another indole moiety and dehydration to form intermediate **D**. Finally, intermediate **C** after hydrogen remove formed the bis-indolylindane-1,3-dione, 2-(1′,3′-dihydro-1H-[2,3']biindolyl-2′-ylidene)-indan-1,3-diones **3(a–e)** (Scheme 2).

**Scheme 3:**
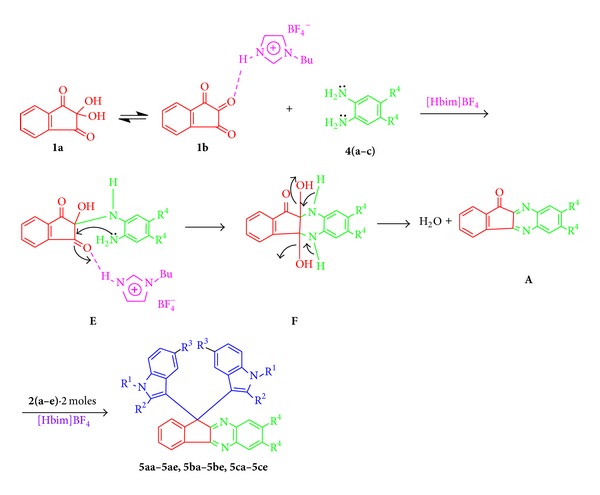
Plausible mechanism for the synthesis of indene-1,3(2H)-denies in the presence of [Hbim]BF_4_ ionic medium.

In this case, initially the condensation of ninhydrin (**1**) and 1,2-phenylenediamine **4(a–c)** took place to produce the intermediate **E→F→A**, which reacted with 2 mol of indoles **2(a–e)** via the intermediate **A** to generate **5aa–5ae**, **5ba–5be**, **5ca–5ce** in high yield (Table 2, entries 1-15) (Scheme 3).

**Scheme 4:**
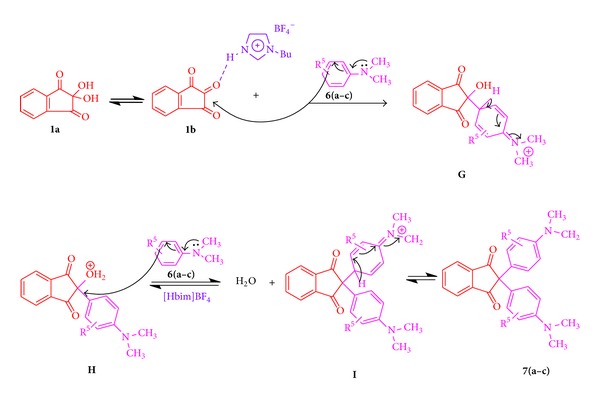
Plausible mechanism for the synthesis of 2,2-bis(4-(dimethylamino)phenyl)-1H-indene-1,3(2H)-denies in the presence of [Hbim]BF_4_ ionic medium.

Reaction ninhydrin (**1**) with different substituted N, N-dimethyl aniline **6(a–c)** via intermediates transformation **G→H→I**, finally with hydrogen removes and aromatization to produce **7(a–c)** (Scheme 4).

**Figure 1:**
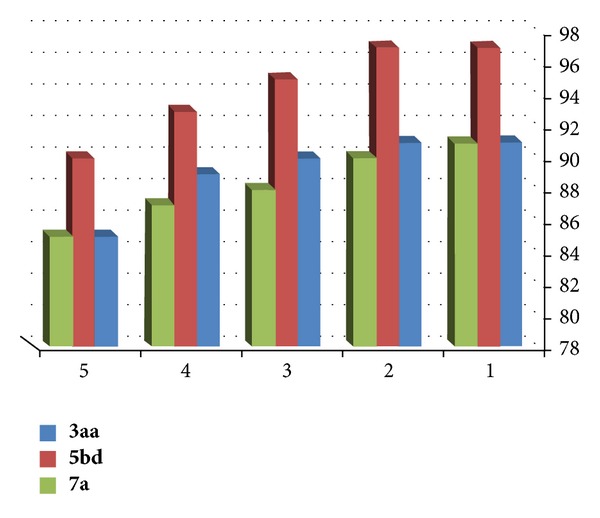
Recyclability of [Hbim]BF_4_ ionic liquid as catalyst.

**Figure 2:**
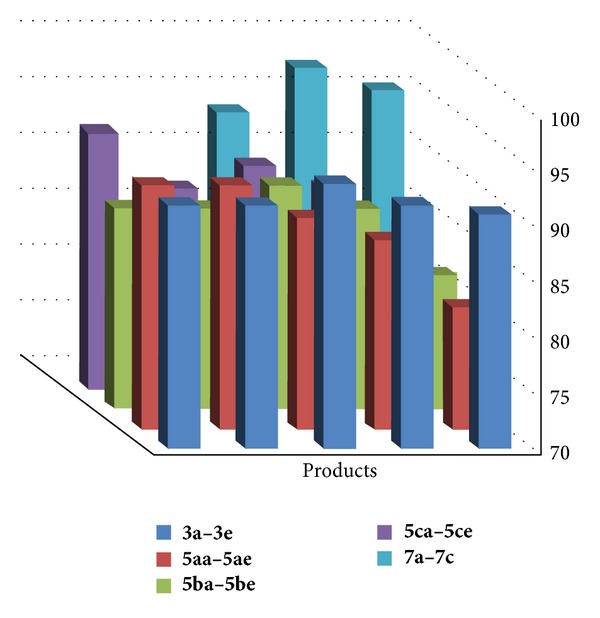
Atomic economy of products.

We also investigated the recycling of the ionic liquid [Hbim]BF_4_ under solvent free conditions. The reusability of IL was tested using a model reaction of ninhydrin and insole, 4,5-dimethylbenzene-1,2-domain and 2-methyl-1H-indole, and N,N-dimethylaniline as substrates for preparation of **3aa**, **5bd**, and **7a**, respectively. After completion of the reaction, the reaction mixture was filtered to isolate the desired IL which was washed with ethyl acetate in order to remove the impurities and unreacted substrates and used for the next run. It was observed that there was no any substantial loss of catalytic activity even after the fifth run as indicated in Figure 1. The greenness of the protocols can be easily proven using the concept atom economy. Thus, we investigated the atom economy for each derivative synthesized and listed the values in Tables 1, 2, and 3 (Figure 2) (see Supplementary data available online at http://dx.doi.org/10.1155/2013/528329). From the values, it is clearly seen that the protocols are atom economy and generate the least amount of waste which is a complimentary ecofriendly aspect of catalyst. The results show that present ionic liquids such as [Hbim]BF_4_ are efficient catalyst with respect to the low reaction times and the high yields.

## 4. Conclusion

In summary, we describe a novel use of ionic liquids for the synthesis of an efficient synthesis of bis-indolylindane-1,3-dione, 2-(1′,3′-dihydro-1H-[2,3′]biindolyl-2′-ylidene)-indan-1,3-diones, and 2,2-bis(4-(dimethylamino)phenyl)-1H-indene-1,3(2H)-denies using [Hbim]BF_4_ ionic medium in excellent yields. The notable features of this procedure are high conversions, operational simplicity, good reaction rates, clean reaction profiles, and ease of isolation of products, which make this process quite simple, convenient, and environmentally benign for the synthesized compounds.

## Supplementary Material

Atom economy (atom efficiency) describes the conversion efficiency of a chemical process in terms of all atoms involved (desired products produced). In an ideal chemical process, the amount of starting materials or reactants equals the amount of all products generated (see stoichiometry) and no atom is wasted. Atom economy is an important concept of green chemistry philosophy, and one of the most widely used ways to measure the “greenness” of a process or synthesis.

## Figures and Tables

**Table 1:** Synthesis of bis-indolylindane-1,3-dione, 2-(1′,3′-dihydro-1H-[2,3′]biindolyl-2′-ylidene)-indan-1,3-diones in the presence of [Hbim]BF_4_ ionic medium.

Entry	R^1^	R^2^	R^3^	Product	Time (min)	Yield (%)^a^	Melting point	
							Report, m.p. (°C)
1	H	H	H	**3a**	5	95	207–209	208–210 [9]
2	H	H	F	**3b**	10	90	121–123	Prepared for the first time
3	H	H	Br	**3c**	8	93	105–107	104–106 [9]
4	H	CH_3_	H	**3d**	4	97	107–109	108–110 [9]
5	CH_3_	H	H	**3e**	3	97	233–235	232–234 [9]

^a^Yield refers to pure products after crystallization.

**Table 2:** Preparation of **5** in ionic liquid [Hbim]BF_4_.

Entry	R^1^	R^2^	R^3^	R^4^	Product	Time (min)	Yield (%)^a^	Melting Point	
								Report, m.p. (°C)	Li. m.p. (°C) [Ref]
1	H	H	H	H	**5aa**	10	93	277–279	276–278 [9]
2	H	H	F	H	**5ab**	12	90	224–226	Prepared for the first time
3	H	H	Br	H	**5ac**	8	92	275–277	274–276 [9]
4	H	CH_3_	H	H	**5ad**	7	94	195–197	Prepared for the first time
5	CH_3_	H	H	H	**5ae**	5	95	183–185	182–184 [9]
6	H	H	H	CH_3_	**5ba**	7	93	217–219	218–220 [9]
7	H	H	F	CH_3_	**5bb**	7	92	210–212	Prepared for the first time
8	H	H	Br	CH_3_	**5bc**	7	95	219–221	Prepared for the first time
9	H	CH_3_	H	CH_3_	**5bd**	5	97	205–207	204–206 [9]
10	CH_3_	H	H	CH_3_	**5be**	3	95	171–173	170–172 [9]
11	H	H	H	Cl	**5ca**	10	94	228–230	Prepared for the first time
12	H	H	F	Cl	**5cb**	15	91	199–201	Prepared for the first time
13	H	H	Br	Cl	**5cc**	8	93	223–225	Prepared for the first time
14	H	CH_3_	H	Cl	**5cd**	7	94	253–255	Prepared for the first time
15	CH_3_	H	H	Cl	**5ce**	5	95	185–187	186–188 [9]

^a^Yield refers to pure products after crystallization.

**Table 3:** Preparation of **7** in ionic liquid [Hbim]BF_4_.

Entry	R^5^	Product	Time (min)	Yield (%)^a^	Melting point (°C)
1	H	**7a**	15	95	New
2	*m*-CH_3_	**7b**	5	97	New
3	*m*-Cl	**7c**	10	93	New

^a^Yield refers to pure products after crystallization.
