# Supplementary material for: An Efficient Synthesis of Bis-indolylindane-1,3-diones, Indan-1,3-diones, and Indene-1,3(2H)-denies Using [Hbim]BF4 Ionic Medium
Source: ISRN Org Chem. 2013 Dec 16;2013:528329. doi: 10.1155/2013/528329 (PMC3876826; doi:10.1155/2013/528329)
Supplement: Supplementary file 1 — Atom economy (atom efficiency) describes the conversion efficiency of a chemical process in terms of all atoms involved (desired products produced). In an ideal chemical process, the amount of starting materials or reactants equals the amount of all products generated (see stoichiometry) and no atom is wasted. Atom economy is an important concept of green chemistry philosophy, and one of the most widely used ways to measure the “greenness” of a process or synthesis. [file 528329.f1.doc]

**Supplementary Data**


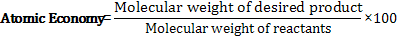


**Table 1.** Atomic economy of bis-indolylindane-1,3-dione, 2-(1',3'-dihydro-1H-[2,3']biindolyl-2'-ylidene)-indan-1,3-diones

| AE | Reactant (s) | Product | Entry |
| --- | --- | --- | --- |
| 91 |  | **2a** | 1 |
| 92 |  | **2b** | 2 |
| 94 |  | **2c** | 3 |
| 92 |  | **2**d | 4 |
| 92 |  | **2e** | 5 |

**Table 2.** Atomic economy of , bisindolylindeno[1,2-b]quinoxalines

| AE | Reactant (s) | Product | Entry |
| --- | --- | --- | --- |
| 81 |  | **5aa** | 1 |
| 87 |  | **5ab** | 2 |
| 89 |  | **5ac** | 3 |
| 92 |  | **5ad** | 4 |
| 92 |  | **5ae** | 5 |
| 82 |  | **5ba** | 6 |
| 88 |  | **5bb** | 7 |
| 90 |  | **5bc** | 8 |
| 88 |  | **5bd** | 9 |
| 88 |  | **5be** | 10 |
| 83 |  | **5ca** | 11 |
| 88 |  | **5cb** | 12 |
| 90 |  | **5cc** | 13 |
| 88 |  | **5cd** | 14 |
| 93 |  | **5ce** | 15 |

**Table 3.** Atomic economy of and 2,2-bis(4-(dimethylamino)phenyl)-1H-indene-1,3(2H)-denies

| AE | Reactant (s) | Product | Entry |
| --- | --- | --- | --- |
| 91 |  | **2a** | 1 |
| 92 |  | **2b** | 2 |
| 93 |  | **2c** | 3 |
